# Comparing anti-tumor and anti-self immunity in a patient with melanoma receiving immune checkpoint blockade

**DOI:** 10.1186/s12967-024-04973-7

**Published:** 2024-03-05

**Authors:** Shuming Chen, Tracee L. McMiller, Abha Soni, Farah Succaria, John-William Sidhom, Laura C. Cappelli, Livia A. Casciola-Rosen, Isaac R. Morales, Preethi Sankaran, Alan E. Berger, Julie Stein Deutsch, Qingfeng C. Zhu, Robert A. Anders, Jody E. Hooper, Drew M. Pardoll, Evan J. Lipson, Janis M. Taube, Suzanne L. Topalian

**Affiliations:** 1 Department of Surgery, Johns Hopkins University School of Medicine, Baltimore, MD 21287 USA; 2 Department of Dermatology, Johns Hopkins University School of Medicine, Baltimore, MD 21287 USA; 3 Department of Oncology, Johns Hopkins University School of Medicine, Baltimore, MD 21287 USA; 4 Department of Biomedical Engineering, Johns Hopkins University School of Medicine, Baltimore, MD 21287 USA; 5 Department of Medicine, Johns Hopkins University School of Medicine, Baltimore, MD 21287 USA; 6 Department of Pathology, Johns Hopkins University School of Medicine, Baltimore, MD 21287 USA; 7 Bloomberg~Kimmel Institute for Cancer Immunotherapy, Johns Hopkins University School of Medicine, Baltimore, MD 21287 USA; 8 Present Address: Contra Costa Pathology Associates, Pleasant Hill, CA USA; 9 Present Address: Mount Sinai School of Medicine, New York, NY USA; 10 Present Address: Crossbow Therapeutics, Cambridge, MA USA; 11 Present Address: Stanford University School of Medicine, Palo Alto, CA USA

**Keywords:** COX-2, Gene expression profiling (GEP), Immune checkpoint blockade (ICB), Immune related adverse events (irAEs), Melanoma, Rapid autopsy, Tumor infiltrating lymphocytes (TILs)

## Abstract

**Background:**

Tumor regression following immune checkpoint blockade (ICB) is often associated with immune-related adverse events (irAEs), marked by inflammation in non-cancerous tissues. This study was undertaken to investigate the functional relationship between anti-tumor and anti-self immunity, to facilitate irAE management while promoting anti-tumor immunity.

**Methods:**

Multiple biopsies from tumor and inflamed tissues were collected from a patient with melanoma experiencing both tumor regression and irAEs on ICB, who underwent rapid autopsy. Immune cells infiltrating melanoma lesions and inflamed normal tissues were subjected to gene expression profiling with multiplex qRT-PCR for 122 candidate genes. Subsequently, immunohistochemistry was conducted to assess the expression of 14 candidate markers of immune cell subsets and checkpoints. TCR-beta sequencing was used to explore T cell clonal repertoires across specimens.

**Results:**

While genes involved in MHC I/II antigen presentation, IFN signaling, innate immunity and immunosuppression were abundantly expressed across specimens, irAE tissues over-expressed certain genes associated with immunosuppression (*CSF1R, IL10RA, IL27/EBI3, FOXP3, KLRG1, SOCS1, TGFB1*), including those in the COX-2/PGE2 pathway (*IL1B, PTGER1/EP1* and *PTGER4/EP4*). Immunohistochemistry revealed similar proportions of immunosuppressive cell subsets and checkpoint molecules across samples. TCRseq did not indicate common TCR repertoires across tumor and inflammation sites, arguing against shared antigen recognition between anti-tumor and anti-self immunity in this patient.

**Conclusions:**

This comprehensive study of a single patient with melanoma experiencing both tumor regression and irAEs on ICB explores the immune landscape across these tissues, revealing similarities between anti-tumor and anti-self immunity. Further, it highlights expression of the COX-2/PGE2 pathway, which is known to be immunosuppressive and potentially mediates ICB resistance. Ongoing clinical trials of COX-2/PGE2 pathway inhibitors targeting the major COX-2 inducer IL-1B, COX-2 itself, or the PGE2 receptors EP2 and EP4 present new opportunities to promote anti-tumor activity, but may also have the potential to enhance the severity of ICB-induced irAEs.

**Supplementary Information:**

The online version contains supplementary material available at 10.1186/s12967-024-04973-7.

## Background

Several decades ago, an association was established between anti-tumor and anti-self immunity when it was discovered that normal melanosomal self-antigens expressed by melanomas could elicit anti-melanoma immunity [1]. Later it was appreciated that both anti-self and anti-tumor immunity are regulated by shared mechanisms, including the PD-1 and CTLA-4 immune checkpoint pathways. These pathways that normally maintain self-tolerance can be exploited by cancer cells to evade immune attack. Consequently, disrupting these pathways through the application of immune checkpoint blockade (ICB) has emerged as an effective approach in cancer treatment [2], but is often associated with undesired inflammation in normal tissues.

Due to its broad profile of anti-cancer activity, ICB has become a cornerstone of oncology. Nevertheless, in ~ 15–20% of patients, anti-PD-(L)1 monotherapy causes the emergence of severe adverse events that are often immune-related (irAEs). Such events are significantly more frequent in patients receiving combination ICB with anti-PD-1 plus anti-CTLA-4. ICB’s anti-cancer efficacy is strongly associated with its induction of irAEs [3], sparking interest in exploring two closely-related areas: first, the potential immunological mechanisms underlying this association; and second, the effective management of irAEs without compromising anti-tumor efficacy. Hypothetically, the association between ICB-induced anti-tumor efficacy and irAEs could stem from two mechanisms, perceived as parallel or linked processes. In a parallel process, ICB would activate T-cell clones with distinct specificities that separately mediate anti-tumor activity or irAEs. In contrast, in a linked process, individual ICB-activated T-cell clones would recognize antigens shared by both tumor and normal cells. Which mechanism predominates, or whether they co-exist, is currently unknown.

To understand the functional relationship between anti-tumor efficacy and irAEs mediated by ICB, we leveraged a rapid autopsy approach to gather multiple tissue samples from a single patient with advanced melanoma who experienced both tumor regression and irAEs during treatment with anti-PD-1 and anti-CTLA-4. In this exploratory study, we employed gene expression profiling (GEP) and immunohistochemistry (IHC) to evaluate the expression of candidate immune cell subset and regulatory markers, and T-cell receptor sequencing (TCRseq) to query the relatedness of the T-cell repertoires in geographic regions of immune cell infiltrates within tumor deposits and inflamed non-cancerous tissues. These studies revealed remarkable similarities between tumors and normal inflamed tissues, including substantial expression of the immunosuppressive cyclo-oxygenase 2/prostaglandin E2 (COX-2/PGE2) pathway. Further, they suggested that distinct T-cell repertoires were involved in anti-tumor vs anti-self reactivity.

## Materials and methods

### Rapid autopsy and tissue processing

**Fig. 1:**
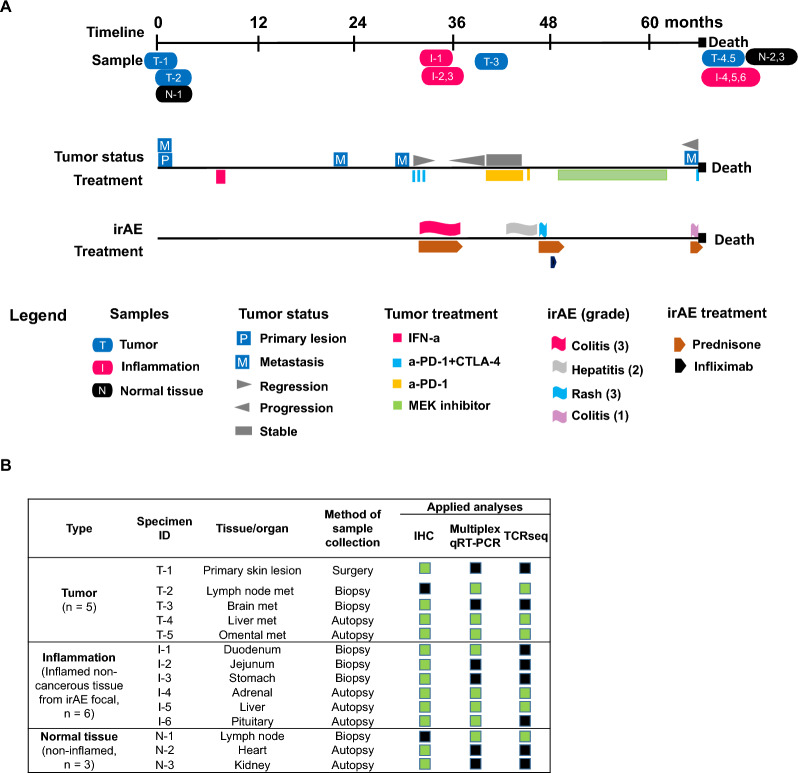
Clinical timeline and tissue specimens. **A**, Melanoma course, occurrence of irAEs, and specimens collected from patient MA-6. The top timeline shows tissue collections; the middle timeline shows melanoma status and systemic therapies (not including focal radiotherapy); the bottom timeline shows the irAEs and their pharmacologic management. Not included in the bottom timeline is the onset of grade 2 adrenal insufficiency of clinically unclear etiology, treated with hydrocortisone replacement. IFN-a, adjuvant interferon alfa. **B**, Tissue specimens from patient MA-6 that were used in this study. Biopsy and surgical specimens were collected pre-mortem, others were collected at autopsy. Green boxes, assays performed; black boxes, assays not done. *IHC* immunohistochemistry; met, metastasis, *qRT-PCR* quantitative real-time reverse transcription polymerase chain reaction, *TCRseq* T-cell receptor sequencing

Tissues were collected from a consenting patient (“MA-6”) enrolled on the Legacy Gift Rapid Autopsy Program at Johns Hopkins University School of Medicine, approved by the Hopkins Institutional Review Board, as previously described [4]. Premortem biopsy specimens preserved as formalin-fixed paraffin-embedded (FFPE) blocks were obtained from pathology archives, including the primary melanoma lesion, melanoma metastases to lymph nodes and brain, and inflamed non-cancerous tissues from the stomach, duodenum, and jejunum. A rapid autopsy was conducted within several hours after death, yielding additional melanoma metastases from the liver and omentum, inflamed non-cancerous regions of the liver, adrenal gland and pituitary gland, and selected normal tissue controls (heart, kidney) (Fig. 1). These samples were preserved as FFPE blocks and evaluated microscopically with hematoxylin and eosin (H&E) staining for structural preservation, cell integrity, and tumor cellularity. Additionally, following informed consent, blood was collected at several pre-mortem intervals and at autopsy for serum or plasma preparation.

### Laser capture microdissection (LCM) and macrodissection

**Fig. 2:**
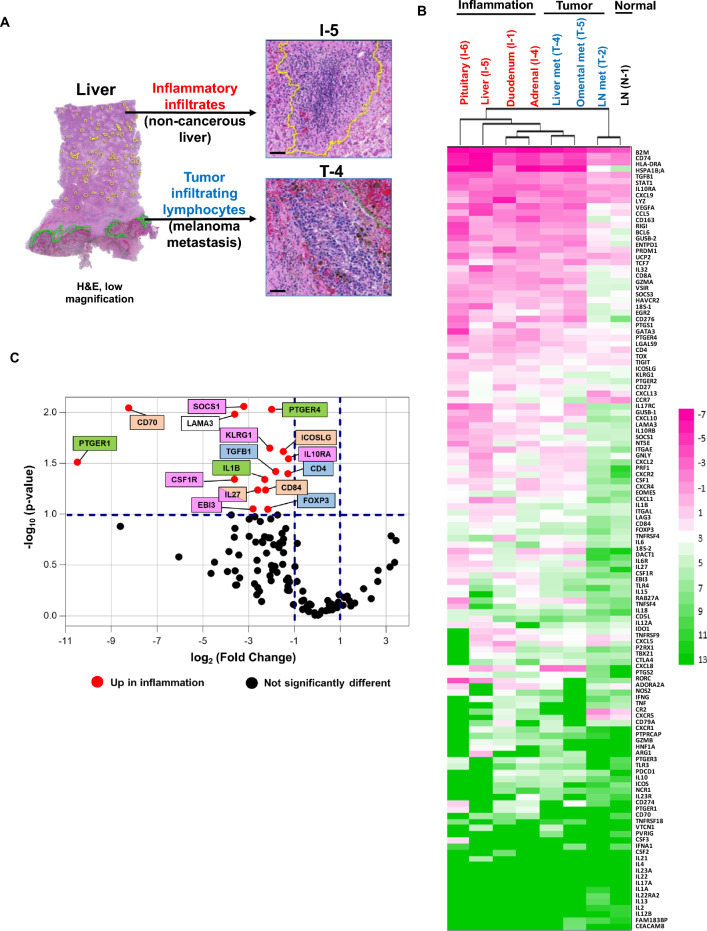
Similarities and differences in gene expression by immune cells infiltrating inflamed tissues vs melanoma metastases. **A**, LCM of immune cells infiltrating regions of normal tissue inflammation and cancer in a post-mortem liver specimen. Yellow circles, dissected areas containing inflammatory infiltrates in the non-cancerous portion of the liver (specimen I-5); green circles, dissected areas containing tumor infiltrating immune cells in a metastatic melanoma deposit (specimen T-4). H&E, hematoxylin and eosin staining. Black bars, 100 µm. **B**, Unsupervised hierarchical clustering of *PTPRC*-normalized Ct values reveals similarities in expression of many genes across all samples. Expression of 122 candidate genes was evaluated with multiplex qRT-PCR. *PTPRC-*normalized Cts (Ct_gene_ – Ct_PTPRC_) were clustered and visualized by heat map using Java TreeView. Pink or green colors indicate genes expressed abundantly or poorly, respectively, in the samples. Clustering reveals a high degree of similarity between a lymph node metastasis and a normal lymph node specimen, but does not differentiate the 6 remaining specimens including 2 melanoma metastases and 4 non-cancerous inflamed tissues. **C**, Volcano plot reveals groups of genes with related functions upregulated in 4 inflamed normal tissues vs 3 tumor specimens. *PTPRC*-normalized Ct values were used to calculate fold changes of gene expression. Red dots in the upper left region of the plot indicate genes significantly upregulated (fold change magnitude ≥ 2.0, and Welch’s t-test 2-sided p-value ≤ 0.10) in inflamed normal tissues. Genes related to immunosuppressive cell types and cytokines are indicated by magenta boxes, Tregs by blue boxes, COX-2/PGE2 pathway by green boxes, and lymphocyte activation by peach boxes. Black dots in the lower half of the plot indicate genes without differential expression. No genes were upregulated in tumor samples. Genes that were undetectable in any specimen (*IL4, IL17A, IL22,* and *IL23A*) were omitted

FFPE blocks were sectioned into 7-µm thick slices on Arcturus PEN membrane glass slides for LCM (Applied Biosystems, Foster City, CA). A serial 5 µm-thick section was stained with H&E to assess the geography of immune cell infiltrates. To collect immune infiltrates for RNA and DNA isolation, LCM was conducted using a Leica LMD6000 microscope (Leica Camera, Wetzlar, Germany). From tumor samples, infiltrating immune cells (lymphocytes, myeloid cells) abutting viable tumor cells were excised, avoiding necrotic areas. From non-cancerous inflamed tissues and normal tissues, lymphoid/myeloid infiltrates were dissected (Fig. 2A). To insure adequate amounts of tissue for RNA and DNA extraction, hundreds of microscopic areas were sometimes collected per specimen from consecutive slides, since in some cases small patches of immune infiltrates were widely separated by cancerous or normal tissue areas (Fig. 2A). Additionally, manual scalpel macrodissection of microscopically-defined areas from an inflamed non-cancerous adrenal gland specimen was performed on marked FFPE slides under visual inspection to obtain sufficient DNA for TCRseq analysis.

### RNA extraction and multiplex real-time quantitative reverse transcription polymerase chain reaction (qRT-PCR)

RNA extraction, reverse transcription, and qRT-PCR with pre-amplification were performed using published methods [5]. The integrity of mRNAs was confirmed by robust amplification of transcripts from several housekeeping genes including *18S, ACTB, GAPDH, GUSB* and *PTPRC* (data not shown). Short amplicons (~ 100 bp) were consistently used to optimize qRT-PCR efficiency in RNAs extracted from FFPE specimens, as previously reported [5].

Multiplex qRT-PCR employed custom-made TaqMan Low-Density Array (TLDA) Micro Fluidic Cards (Applied Biosystems), with which PCR was conducted for 45 cycles. TLDA cards contained triplicate reaction wells for each of 122 candidate genes and three endogenous control genes (*18S, GUSB, PTPRC*) (Additional file 1: Table S1). Average cycle threshold (Ct) values were normalized using either *PTPRC* (CD45, pan leukocyte marker) or *GUSB* (a cell lineage non-specific housekeeping gene). Undetermined Ct values (here defined as > 40) were adjusted to 40 for analysis. Fold changes in gene expression between tumor and inflammation samples were determined using the 2^−ΔΔCt^ method. Volcano plots, combining information on fold expression differences and p-values (determined using a two-sided Welch’s t-test), were created using GraphPad (Somerville, MA). To generate heat maps based on ∆Ct values, unsupervised clustering was performed using Cluster 3.0 with a city-block distance metric and average linkage. The heat map was then visualized using Java TreeView Version 1.1.6r4.

### IHC analysis

Serial 5 µm FFPE sections obtained from 12 tissue specimens from patient MA-6, including tumor (n = 4), inflammation (n = 6), and normal tissue controls (n = 2), were stained with H&E and antibodies specific for 14 candidate immune-related markers to detect cellular subsets (CD3, CD4, CD8, CD79a [B cells], CD163 [M2 macrophages], FOXP3 [Tregs], RORgt [Th17 transcription factor]), immune regulatory pathways (CSF-1R, IDO-1, LAG-3, PD-1, PD-L1), and inflammation (C4d [C4 degradation product, a classical and lectin complement pathway activation marker] and COX-2). A non-specific mouse-anti-human IgG was used as an isotype staining control. IHC was conducted as described [6], with the exception of C4d staining, which was performed with routine automated techniques by the Immunopathology Laboratory at Johns Hopkins Medical Institutions. Expression of C4d, COX-2, and PD-L1 was assessed visually for patterns (diffuse, focal, and patchy), and semi-quantitative expression scores of 0 to 3 + were assigned. Expression of the remaining 11 markers was quantified as cell density (number of positive cells per mm^2^ tissue area) using HALO software (Indica Labs, Albuquerque, NM) as described [6]. Areas of immune cell infiltrates in tumor or inflamed normal tissues, excluding areas of necrosis and normal tissue background, were annotated. Enumeration of cell phenotypes was performed using Halo software with visual verification of algorithm performance. The density of positive cells for each marker was compared between tumor and inflammation samples. Statistical analysis was performed using the Wilcoxon rank-sum test via the "wilcox_test" function from the R ‘‘coin’’ package (version 1.2–2) with the distribution set to ‘‘exact.’’ All p-values are two-sided.

### Serologic analysis

Sera or plasma prepared from blood collected from patient MA-6 before and during ICB treatment and at autopsy were assayed for novel antibodies with an established immunoprecipitation platform [7]. Sera were used to precipitate ^35^S-methionine-labeled lysates made from cultured HeLa cells, expressing a large portion of the human proteome, or 624mel cells expressing shared non-mutated melanoma antigens (e.g., MAGE-A3, tyrosinase, MART-1/Melan-A, gp75, gp100). Immunoprecipitates were electrophoresed on SDS–polyacrylamide gels and visualized by fluorography. Additionally, a commercial line immunoblot assay (EUROLine, EUROIMMUN, Germany) was used to assess 43 defined antibody specificities associated with scleroderma, myositis, and systemic lupus erythematosus (SLE).

### DNA extraction and TCRseq

Genomic DNA (gDNA) was extracted from FFPE tissue samples using the QIAamp DNA FFPE Tissue Kit (QIAGEN, Hilden, Germany). The concentration and purity of gDNA were assessed using a NanoDrop spectrophotometer (Thermo Fisher). TCRB sequencing and analysis were conducted by Adaptive Biotechnologies (Seattle, WA) using their ImmunoSEQ platform. Sequencing data were exported from ImmunoSEQ ANALYSES 3 and analyzed using DeepTCR [8], a collection of algorithms that utilize deep learning techniques to learn the underlying structural distribution of T-cell repertoire. We used the variational autoencoder algorithm within DeepTCR to characterize the repertoire in an unsupervised fashion. The algorithm takes in all TCR sequences (at the amino acid level) that were seen in any lesion of the patient and learns a latent representation to describe the sequences. This latent representation consists of 256 structural concepts that have been learned within the data. After each sequence has been re-represented by these 256 structural features, a weighted average of the feature can be computed for a given sample. These values per sample were used to construct a clustermap, allowing us to compare the structural relatedness of various samples collected in this study. Only productive TCR clones were included in the analysis, and template counts were summarized for nucleotide sequences that translated into the same amino acid sequence.

### Cell cultures and COX-2/PGE2 detection

Nineteen human tumor cell lines representing six different histologies (melanoma: 397mel, 537mel, 624mel, 888mel, 1011mel, 1102mel, 1558mel; renal cell carcinoma: 2192R, 2193R, 786-O, ACHN; head and neck squamous cell carcinoma: JHU-011, JHU-022, JHU-029; gastric cancer: AGS, NCI-N87; colorectal cancer: HT-29, SW620; non-small-cell lung cancer: A549) were derived and maintained as described [9]. Monocytes were enriched from cryopreserved normal donor peripheral blood mononuclear cells (PBMC) and cultured as described [9]. Cultured tumor cells and monocytes were treated with recombinant human cytokines, including IFN-g 100 IU/ml (Biogen, Cambridge, MA); IL-1A 10 ng/ml, IL-1B 10 ng/ml, IL-17A 50 ng/ml, IL-32-g 100 ng/ml (R&D Systems, Minneapolis, MN); and TNF-a 100 U/ml (Genentech, Washington, DC), for 1 day as described [9].

COX-2 protein was detected in cultured cells using Western blotting and intracellular flow cytometry according to standard protocols. For Western blotting, antibodies against COX-2 (clone D5H5) and COX-1 (clone D2G6) were obtained from Cell Signaling (Beverly, MA). Beta-actin (clone AC-15, Sigma, Israel) was detected as a loading control. Target molecule density was quantified using the ImageJ program (https://imagej.nih.gov/ij/download.html) and normalized to the density of beta-actin, then further normalized by constitutive COX-2 expression in an indicator cell line, JHU-011. COX-2 was also detected by intracellular flow cytometry (clone AS67, Becton Dickinson, Franklin Lakes, NJ) and data were analyzed using FlowJo software. The expression level of a molecule was calculated as the delta mean fluorescence intensity (∆MFI), obtained by subtracting the MFI of isotype control staining from the MFI of specific staining.

PGE2 was detected in cell culture supernatants by ELISA (Cayman Chemical, Ann Arbor, MI) following the manufacturer's protocol. Supernatants from IL-1B-treated 537mel were used as a PGE2 positive control in every assay. Absorbance at 414 nm was read with the Varioskan Lux microplate reader and analyzed by SkanIt^™^ RE v6.1.1 (ThermoFisher Scientific, Waltham, MA). Very low concentrations of PGE2 sometimes detected in the sera used in cell culture media were subtracted from cell culture supernatant values. Correlation of COX-2 expression and PGE2 secretion was assessed using the Pearson correlation test with 2-sided p-values.

## Results and discussion

### Clinical history and tissue specimens

Patient MA-6, an adult with melanoma and diet-controlled clinically inactive celiac disease, enrolled in the Johns Hopkins Legacy Gift Rapid Autopsy Program before dying in 2017 from widespread stage IV melanoma. Patient MA-6 received multiple immune-based and kinase inhibitor therapies and developed several irAEs (Fig. 1). In 2011, patient MA-6 was diagnosed with a primary cutaneous NRAS-mutant melanoma on the upper back and two metastatic sentinel lymph nodes were resected at that time. Patient MA-6 received adjuvant interferon alfa for ~ 4 weeks, discontinued due to side effects. In 2013, after recurrent cervical lymph node metastases were treated with surgery and radiotherapy, this patient developed further melanoma progression in multiple subcutaneous and intra-abdominal sites. Treatment with nivolumab (anti-PD-1) plus ipilimumab (anti-CTLA-4) resulted in a transient partial tumor regression, accompanied by biopsy-proven grade 3 immune-related enterocolitis requiring prolonged glucocorticoid management. In 2014, melanoma relapse in the brain required two surgeries and radiotherapy. Later that year, melanoma progression at multiple sites occurred, and grade 2 adrenal insufficiency (considered to be an irAE but specific etiology clinically undetermined) necessitated hydrocortisone replacement. Pembrolizumab (anti-PD-1) monotherapy was administered October 2014—February 2015, with melanoma stabilization and grade 2 immune-related hepatitis requiring glucocorticoid management. Then, a single dose of nivolumab (anti-PD-1) caused a steroid-refractory grade 3 rash requiring infliximab (anti-TNF-a). Systemic melanoma therapy with trametinib (MEK inhibitor) from May 2015—August 2016 initially mediated disease stabilization, followed by progression in liver and omental/mesenteric metastases, the latter treated with radiotherapy. A repeat course of nivolumab plus ipilimumab resulted in colitis but was ineffective in controlling melanoma progression, leading to death. At autopsy, tissues were collected 5–8 h post-mortem. Over the 5.7 years from initial melanoma diagnosis to autopsy, 7 pre-mortem and 7 post-mortem tissue specimens were obtained from tumor (n = 5), inflamed organs (n = 6), and non-inflamed normal tissues (n = 3), enabling laboratory assessments to explore the relationship between anti-tumor and anti-self immunity in this patient.

### Comparative gene expression profiling of immune cell infiltrates in tumor vs non-cancerous inflamed tissues

LCM enabled us to precisely dissect areas of immune cell infiltrates in FFPE tissue sections, for comparative gene expression profiling of immune cells infiltrating cancer vs non-cancerous inflamed tissues (Fig. 2A). We employed multiplex qRT-PCR, a robust assay for use with partially degraded RNAs from autopsy specimens, to query 122 candidate genes characterizing immune cell subsets, cytokines/chemokines, and immunomodulatory receptor-ligand pathways (Additional file 1: Table S1). An unsupervised heatmap analysis normalizing gene expression (Ct) values to immune cell content (*PTPRC*, a.k.a. CD45, a pan-immune cell marker) revealed many phenotypic and functional similarities between inflammatory infiltrates in cancerous and non-cancerous tissues (Fig. 2B). This suggested similar immunologic mechanisms underlying anti-tumor and anti-self immunity in patient MA-6. For instance, genes associated with the major histocompatibility complex and antigen presentation (*B2M, CD74, HLA-DRA*), interferon signaling (*CXCL9, STAT1*), immunosuppression (*IL10RA, HAVCR2* [TIM3], *LGALS9* [galectin-9], *TGFB1, TIGIT*), and innate immunity (*RIG1*, *LYZ,*) were abundantly expressed across all samples. Conversely, expression of genes related to neutrophils (*CEACAM8* [CD66b], *CSF2* [GM-CSF]), Th2 cells (*IL4, IL13*) and Th17 cells (*IL17A, IL22, IL22RA2, IL23A*) was low or undetectable across all samples, suggesting that these cell subsets were unlikely to be involved in irAEs or anti-tumor immunity in patient MA-6; the successful detection of these genes with identical assay conditions in other studies in our laboratory diminishes the possibility of technical failure. Consistent with our findings, a dominance of Th1 but not Th17-associated genes has been reported by others in irAE dermatitis and colitis specimens [10].

The B-cell homing chemokine CXCL13, which resides in the IL-21/CXCL13 auto-antibody axis and is also produced by tumor mutation-specific CD8 + TILs in lung cancer [11], exhibited moderate expression across specimens in our study, implying a role for B cells in both anti-tumor and anti-self immunity in this patient.

Sixteen genes were up-regulated in immune infiltrates from non-cancerous inflamed (n = 4) vs tumor (n = 3) tissues (Fig. 2C and Additional file 1: Table S2**;** expression fold change magnitude ≥ 2.0 and p ≤ 0.10 normalized to *PTPRC*). Notably, 12/16 up-regulated genes encode molecules associated with immunosuppressive signaling pathways and cellular subsets, including *CSF1R, IL10RA, IL27/EBI3* [IL-27 heterodimer]*, KLRG1, SOCS1*; Treg hallmarks (*CD4, FOXP3, TGFB1*); and the COX-2/PGE2 pathway (*IL-1B, PTGER1* [EP1, prostaglandin E2 receptor 1], and *PTGER4* [EP4, prostaglandin E2 receptor 4]). Other differentially expressed genes are associated with lymphocyte activation (*CD70, CD84, ICOSLG*). COX-2/*PTGS2*, with known immunosuppressive functions [12], was up-regulated in tumor tissues when expression was normalized to *GUSB* (fold change 4.16, p = 0.09) rather than *PTPRC,* implicating non-immune cells (e.g., tumor and/or stromal cells) as an important source of this enzyme in the tumor microenvironment (TME) (Additional file 2: Figure S1, and Additional file 1: Table S2). Two additional immunosuppressive/pro-carcinogenic factors, *IL6* and *ENTPD1* (CD39), known to be expressed broadly by both immune and non-immune cells such as cancer-associated fibroblasts [13] and endothelial cells [14], were also upregulated in tumor vs inflamed non-cancerous tissues with *GUSB* normalization. The predominantly immunosuppressive gene expression profile up-regulated in non-cancerous inflamed tissues after ICB likely reflects homeostatic feedback inhibition mechanisms that engage after acute immune activation, but may have also dampened anti-tumor immunity in this patient.

### Immunohistochemistry analysis

Following gene expression profiling, IHC was conducted on 12 tissue specimens (4 tumor, 6 inflamed normal tissues, 2 normal controls; Fig. 1B and Additional file 1: Table S3) to further explore the presence of immune cell subsets and expression of immune checkpoint molecules in inflamed non-cancerous tissues compared to tumor deposits. Although there were numerical differences favoring higher densities of marker-positive cells in inflamed normal tissues vs tumor specimens, there were notable similarities in the densities of cells expressing the immune subset markers CD3 (pan T cell), CD4 (T helper), CD8 (cytotoxic T cells), CD163 (M2 macrophages), and RORgt (Th17 transcription factor); and immune modulatory markers including CSF-1R (M2 macrophages), FoxP3 (Tregs), IDO-1 (indolamine-2,3-dioxygenase, immunosuppressive myeloid cells), LAG-3, and PD-1. There was a trend towards increased densities of cells expressing CD79a (B-cell lineage) in inflammation samples (p = 0.10), (Additional file 2: Figure S2). Marker-positive cell densities were generally lower in post-mortem compared to premortem specimens, possibly due to prolonged administration of immunosuppressive drugs to manage irAEs, the different tissue origins of the specimens collected, and/or immune-related effects of prolonged MEK inhibitor administration prior to death, although small group sizes preclude formal statistical analysis. Using the complement C4 degradation product C4d to localize sites of antibody-mediated inflammation, we observed strong diffuse staining of endothelial cells in biopsies from 3 regions of the upper gastrointestinal tract affected by irAEs and underlying celiac disease (stomach, duodenum, jejunum; Additional file 2: Figure S3) but not in other irAE tissues; we also found C4d staining in the primary melanoma lesion, but not in 3 melanoma metastases (Additional file 1: Table S3), indicating that serologic immunity was active in select tissues in this patient. PD-L1 and COX-2, both associated with immunosuppression in the TME, were variably expressed by tumor/epithelial cells and inflammatory cells in both tumor and irAE specimens; as expected from its normal role in gastrointestinal mucosal homeostasis, COX-2 was abundantly expressed by epithelial cells in irAE specimens from the stomach, duodenum and jejunum (Additional file 1: Table S3). Of interest, a heart biopsy taken at autopsy, intended to represent a normal organ, was positive for C4d, COX-2 and PD-L1 staining, suggesting the presence of subclinical inflammation.

Taken together, these results suggest that anti-tumor and anti-self immunity in patient MA-6 receiving ICB utilized shared subsets of immune cells (adaptive and innate) and mechanisms (cellular and serologic) to orchestrate immune responses, and common immune regulatory pathways, consistent with the difficulties thus far in identifying treatment approaches to enhance the therapeutic effects of ICB while protecting against irAEs.

### Serologic analysis

Plasma cell infiltrates and tertiary lymphoid structures have been associated with ICB-mediated tumor regression, suggesting the activation of both serologic and cellular anti-tumor reactivities by ICB [15, 16]. Furthermore, defined serologic reactions have been linked to irAEs in some patients, for instance, elevated anti-acetylcholine receptor antibodies in patients developing myasthenia gravis, or anti-thyroglobulin antibodies in those developing thyroiditis. As mentioned in Results above, in the current study we observed moderate expression of the B cell homing chemokine CXCL13 across tumor and inflammation tissue specimens, C4d deposition on the endothelium of select irAE and tumor tissues, and a borderline increased density of CD79 + B lineage cells in irAE compared to tumor specimens. These results suggested the engagement of serologic reactivities against melanoma and/or normal tissues in patient MA-6. To further explore these findings, we screened 6 serum specimens collected serially from patient MA-6 over 3 years leading to autopsy, for antibodies to proteins expressed in HeLa or 624mel cells. We also assessed these sera for antibodies against well-defined targets of the autoimmune response in scleroderma, myositis, and SLE. This did not reveal preexisting or newly emerging novel or defined serologic reactivities in patient MA-6. In contrast, in similar studies performed with sera from other patients with melanoma receiving ICB, antibodies against cancer-testis antigens were detected in some samples [17]. Of note, the experimental approach taken here would be unable to detect serologic reactivities against mutation-specific melanoma antigens unique to patient MA-6.

### Relatedness of T-cell clones infiltrating inflammation samples

**Fig. 3:**
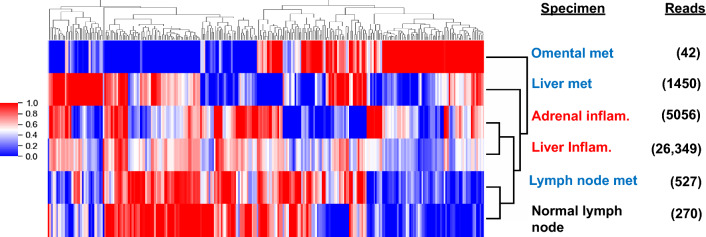
DeepTCR analysis reveals relatedness of T-cell repertoires in two inflammation samples. Summarized counts of productive TCR clones that translate into the same amino acid sequences were used for DeepTCR analysis. Unsupervised clustering of 256 normalized structural concepts is shown. Samples with less than 500 reads should be interpreted with caution. Blue text, tumor samples; red text, inflamed normal tissues

To address whether irAEs induced by ICB in patient MA-6 resulted from cross-reactive T-cell clones across sites of tumor and inflammation, or if different clones were activated by ICB in parallel in these locations, we performed TCRseq and analyzed these data with DeepTCR, a collection of algorithms that utilize deep learning techniques to learn the underlying structural distribution (based on amino acid sequences) of the T-cell repertoire. An unsupervised clustermap revealed the close relatedness of T cells infiltrating both of the inflammation samples examined (adrenal gland and liver), but only a secondary relatedness to metastatic and normal lymph node specimens (Fig. 3). Furthermore, the 3 metastatic melanoma specimens (lymph node, liver, omentum) were not closely related to each other. Overall, the T-cell repertoire mediating irAEs in patient MA-6 appeared to have little overlap with the repertoire of tumor-infiltrating T cells, although our analysis does not exclude the possibility of a small subset of T-cell clones common to both irAE and tumor tissues. These results are in contrast to those reported in two patients with melanoma who developed ICB-related myocarditis, in which common TCR sequences (with undetermined specificities) were found at sites of tumor and myocardial inflammation [18].

A recent study reported that higher abundance of CD4 memory T cells and greater TCR diversity in pre-treatment blood samples, along with changes in TCR clonality in on-treatment blood samples, were associated with the occurrence of more severe irAEs in melanoma patients receiving ICB [19]. These findings highlight the significant role of T cells in the development of irAEs. In view of the preliminary nature of results from the current study, further investigations comparing T-cell clones directly across sites of tumor, irAEs, and peripheral blood in individual patients are necessary to determine the potential relatedness of these T-cell clones. A critical future challenge will be deconvoluting the antigen specificities of T-cell clones found in irAE lesions. The establishment of databases linking TCR sequences with anti-tumor or irAE specificities could also serve as a valuable resource to enhance data interpretation.

### Cytokines found in the TME modulate COX-2 expression and PGE2 secretion in tumor and myeloid cells in vitro

As noted above, with gene expression profiling we observed up-regulation of components of the COX-2/PGE2 pathway in inflammation specimens, including IL-1B (a major inducer of COX-2) and the PGE2 receptors EP1 and EP4; several COX-2 pathway genes were also expressed at lower levels in tumor specimens (Fig. 2B). COX-2 is the pivotal enzyme in the synthesis of the inflammatory mediator PGE2, which interacts with four receptors, EP1-4. COX-2 and PGE2 contribute to a cancer-promoting TME [12] and can confer resistance to ICB in murine in vivo tumor models [20, 21], leading investigators to hypothesize that inhibiting this pathway might improve the anti-cancer efficacy of ICB. Indeed, we have observed up-regulated COX-2 expression in gastric and nasopharyngeal cancers, that generally have low response rates to anti-PD-1 [22, 23], and PGE2-mediated suppression of T cell functions in vitro [24]. In the current study, IHC revealed COX-2 expression in both tumor and immune cells. In irAE specimens, COX-2 was also expressed in gastrointestinal epithelium where it plays a normal homeostatic role (Additional file 1: Table S3).

**Fig. 4:**
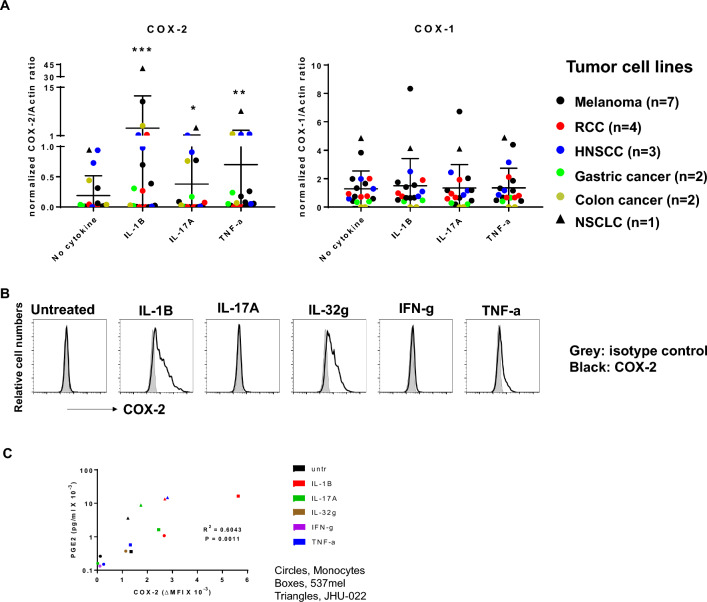
COX-2 expression by tumor and immune cells correlates with PGE2 secretion. **A**, In* human tumor cells, cytokines significantly induce COX-2 but not COX-1 expression across cancer types.* 19 tumor lines from 6 cancer types were cultured with the indicated cytokines for 1 day. COX proteins were detected by Western blotting. Band intensities were quantified and normalized as described in Methods. Means ± SD are indicated. Wilcoxon signed-rank test, 2-sided comparisons to no cytokine, *p = 0.030, **p = 0.005, ***p = 0.001. Similar results were observed with intracellular flow cytometry (not shown). **B**, Cytokine* induction of COX-2 in human monocytes.* Peripheral blood monocytes from a normal donor were exposed to the indicated cytokines in vitro for 1 day. COX-2 expression was assessed by intracellular FACS. Data representative of 2 experiments with monocytes from 3 normal donors. **C**, COX-2 expression by tumor cells and monocytes correlates with PGE2 secretion. 537mel, JHU-022 and monocytes were treated with the indicated cytokines for 1 day. COX-2 expression was quantified by intracellular FACS and PGE2 secretion was measured by ELISA. Pearson correlation, 2-sided p-value

To explore mechanisms regulating COX-2 expression in human tumor and immune cells, we examined constitutive and inducible COX-2 expression in vitro (Fig. 4). TME cytokines that our lab previously found via GEP in melanoma, Hodgkin lymphoma, gastric and nasopharyngeal carcinomas were studied. Among 19 tumor cell lines examined, 6 expressed COX-2 constitutively, and 13 could be induced to express COX-2 after a 1-day exposure to IL-1B, IL-17A, or TNF-a. In contrast, 17 of 19 cell lines constitutively expressed COX-1, and expression was generally stable after cytokine exposure (Fig. 4A and Additional file 2: Figure S4). In peripheral blood monocytes, COX-2 was inducible by IL-1B, IL-32g, and TNF-a, but not by IFN-g or IL-17A (Fig. 4B). We did not detect any COX-2 expression in resting or anti-CD3/CD28-activated T cells (data not shown). Importantly, we found a significant correlation between COX-2 expression in tumor cells or monocytes, and the concentration of PGE2 secreted by those cells into culture supernatants (Fig. 4C). These results suggest that cytokines present within the TME as well as irAE tissues are capable of inducing COX-2 expression in both tumor and myeloid cells, resulting in local PGE2 secretion which may exert immunosuppressive effects in these tissues.

## Conclusions

The implementation of a rapid autopsy protocol allowed the collection of multiple tumor and irAE specimens from patient MA-6 that would otherwise be beyond the scope of conventional biopsies, enabling a comprehensive analysis of this patient to explore the relationship between anti-tumor and anti-self immunity in the context of ICB. The utilization of LCM coupled with quantitative geographic IHC analysis enabled us to focus on immune cell infiltrates in these tissues, revealing similarities between anti-tumor and anti-self immunity, including the substantial expression of the immunosuppressive COX-2/PGE2 pathway. While clinical studies with COX-2 or IL-1B inhibitors have shown benefit in preventing the development of colon and lung cancers, respectively [25, 26], there is not yet evidence that these agents have an impact on established cancers, even when combined with ICB [27–29]. Current early-phase clinical trials testing combinations of anti-PD-(L)1 with specific inhibitors of EP4 alone or EP2 + EP4 in patients with advanced solid tumors (e.g., Clinicaltrials.gov NCT04344795, NCT05205330, NCT05944237) may determine whether anti-tumor and anti-self immunity can be uncoupled using this approach.

### Supplementary Information

**Additional file 1: Table S1.** 122 unique genes included in multiplex qRT-PCR arrays. **Table S2.** Genes differentially expressed in inflamed normal tissue vs. tumor, assessed by multiplex qRT-PCR. **Table S3.** Expression of C4d, COX-2 and PD-L1 detected by immunohistochemistry.

**Additional file 2: Figure S1.** Differentially expressed genes in 4 inflamed normal tissues vs 3 tumor specimens, normalized to *GUSB*. **Figure S2.** IHC did not reveal significant differences in the densities of selected immune cell subsets or cells expressing immune regulatory markers in tumor vs inflamed normal tissue samples. **Figure S3.** Pre-mortem endoscopic jejunal biopsy from patient MA-6, associated with immune-related enteritis on ICB therapy. **Figure S4.** Effect of cytokine exposure on COX-2 expression by 537mel.

## Data Availability

Data are available upon reasonable request.

## Abbreviations

COX-2: Cyclo-oxygenase 2
FFPE: Formalin-fixed paraffin-embedded
gDNA: Genomic DNA
GEP: Gene expression profiling
H&E: Hematoxylin and eosin
ICB: Immune checkpoint blockade
IDO-1: Indolamine-2,3-dioxygenase
IHC: Immunohistochemistry
irAE: Immune-related adverse event
LCM: Laser capture microdissection
PBMC: Peripheral blood mononuclear cells
PGE2: Prostaglandin E2
qRT-PCR: Quantitative reverse transcription polymerase chain reaction
SLE: Systemic lupus erythematosus
TLDA: TaqMan low-density array
TIL: Tumor infiltrating lymphocyte
