# Supplementary material for: Comparing anti-tumor and anti-self immunity in a patient with melanoma receiving immune checkpoint blockade
Source: J Transl Med. 2024 Mar 5;22:241. doi: 10.1186/s12967-024-04973-7 (PMC10916264; doi:10.1186/s12967-024-04973-7)
Supplement: Supplementary file 2 — Additional file 2: Figure S1. Differentially expressed genes in 4 inflamed normal tissues vs 3 tumor specimens, normalized to GUSB. Figure S2. IHC did not reveal significant differences in the densities of selected immune cell subsets or cells expressing immune regulatory markers in tumor vs inflamed normal tissue samples. Figure S3. Pre-mortem endoscopic jejunal biopsy from patient MA-6, associated with immune-related enteritis on ICB therapy. Figure S4. Effect of cytokine exposure on COX-2 expression by 537mel. [file 12967_2024_4973_MOESM2_ESM.pptx]

## Slide 1
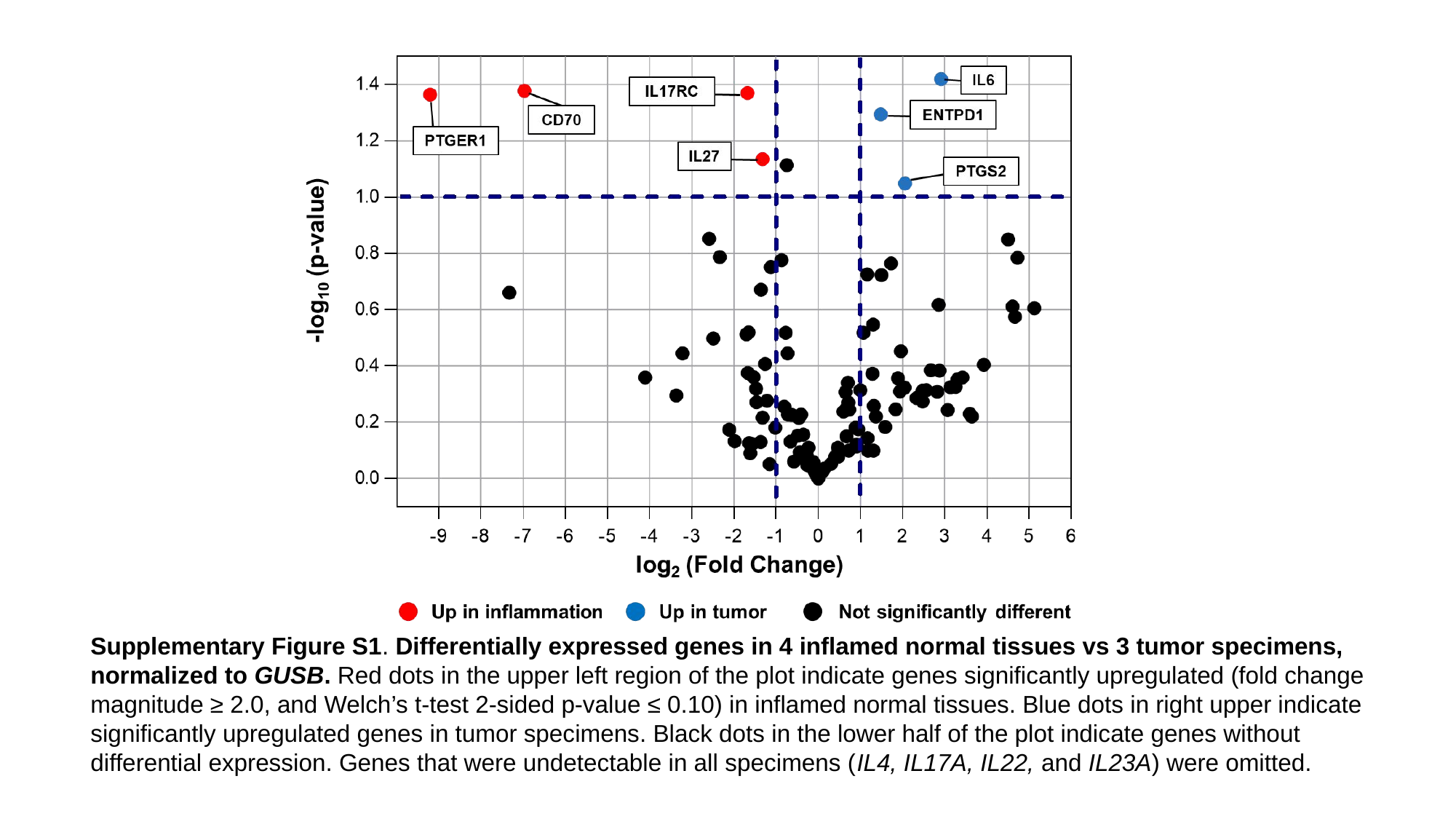

Supplementary Figure S1. Differentially expressed genes in 4 inflamed normal tissues vs 3 tumor specimens, normalized to GUSB. Red dots in the upper left region of the plot indicate genes significantly upregulated (fold change magnitude ≥ 2.0, and Welch’s t-test 2-sided p-value ≤ 0.10) in inflamed normal tissues. Blue dots in right upper indicate significantly upregulated genes in tumor specimens. Black dots in the lower half of the plot indicate genes without differential expression. Genes that were undetectable in all specimens (IL4, IL17A, IL22, and IL23A) were omitted.

## Slide 2
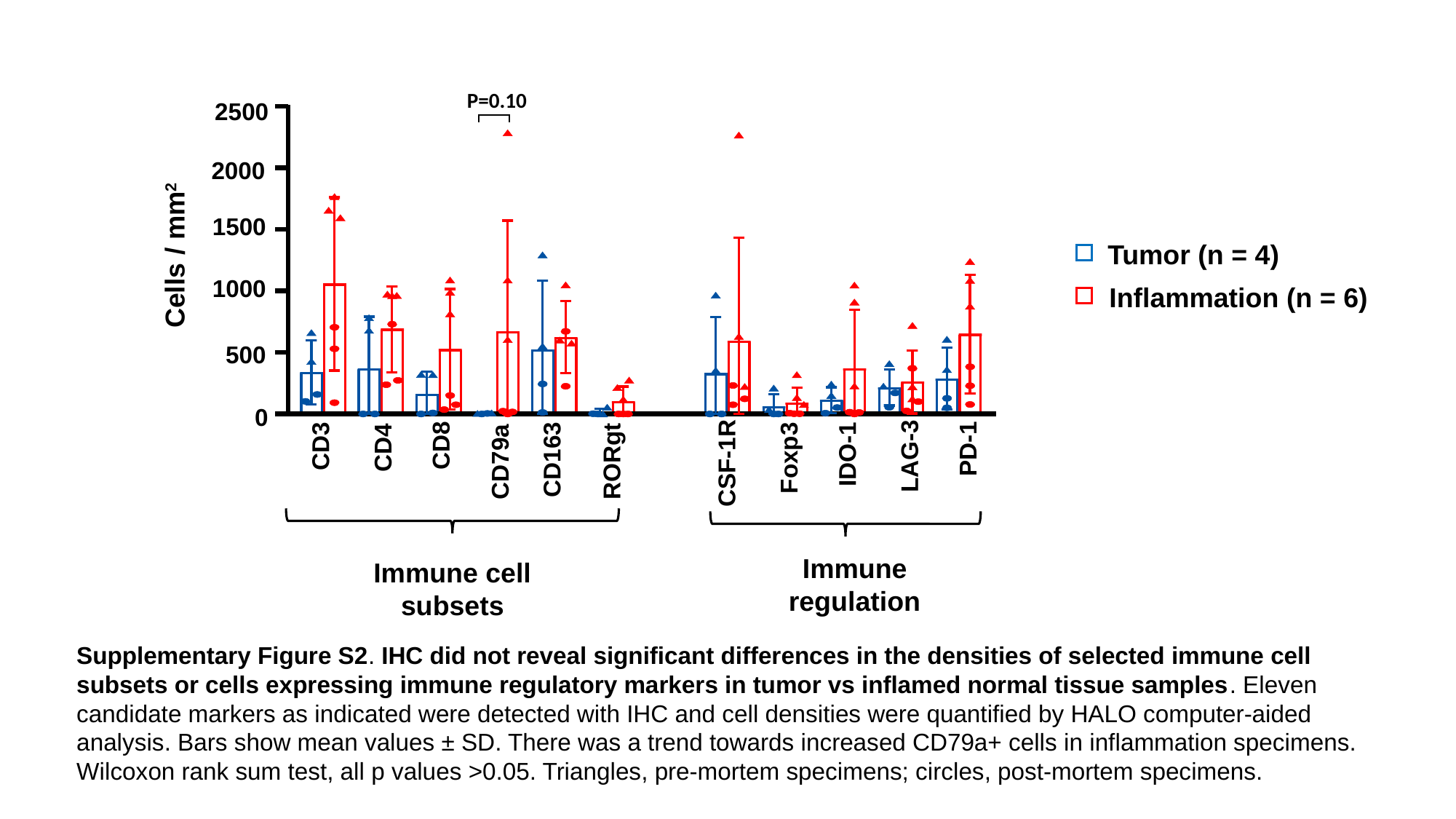

2500
2000
1500
1000
500
0
Tumor (n = 4)
Inflammation (n = 6)
Cells / mm2
CD8
PD-1
CD3
CD4
IDO-1
Foxp3
LAG-3
RORgt
CD163
CD79a
CSF-1R
Immune regulation
Immune cell subsets
P=0.10
Supplementary Figure S2. IHC did not reveal significant differences in the densities of selected immune cell subsets or cells expressing immune regulatory markers in tumor vs inflamed normal tissue samples. Eleven candidate markers as indicated were detected with IHC and cell densities were quantified by HALO computer-aided analysis. Bars show mean values ± SD. There was a trend towards increased CD79a+ cells in inflammation specimens. Wilcoxon rank sum test, all p values >0.05. Triangles, pre-mortem specimens; circles, post-mortem specimens.

## Slide 3
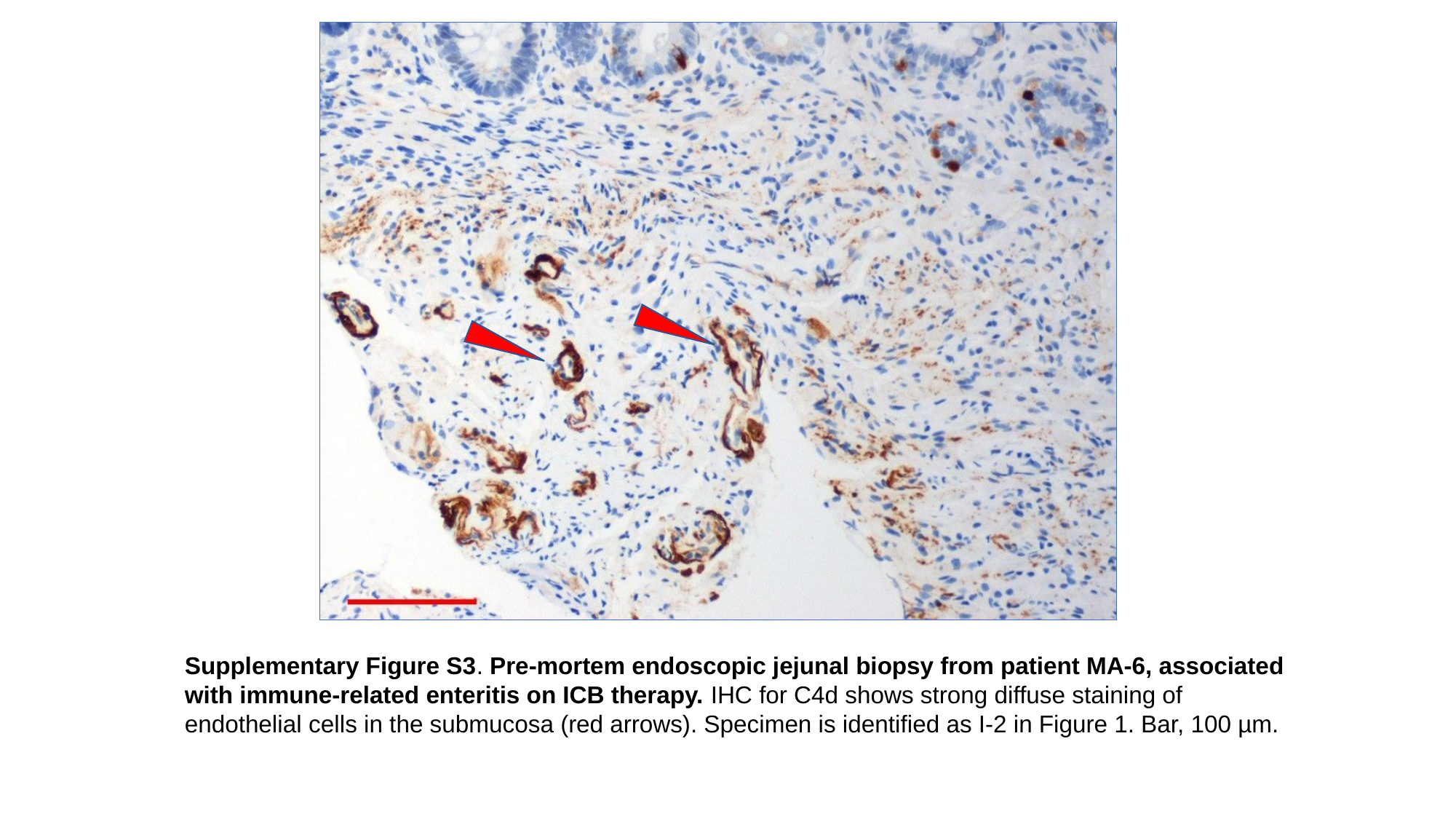

Supplementary Figure S3. Pre-mortem endoscopic jejunal biopsy from patient MA-6, associated with immune-related enteritis on ICB therapy. IHC for C4d shows strong diffuse staining of endothelial cells in the submucosa (red arrows). Specimen is identified as I-2 in Figure 1. Bar, 100 µm.

## Slide 4
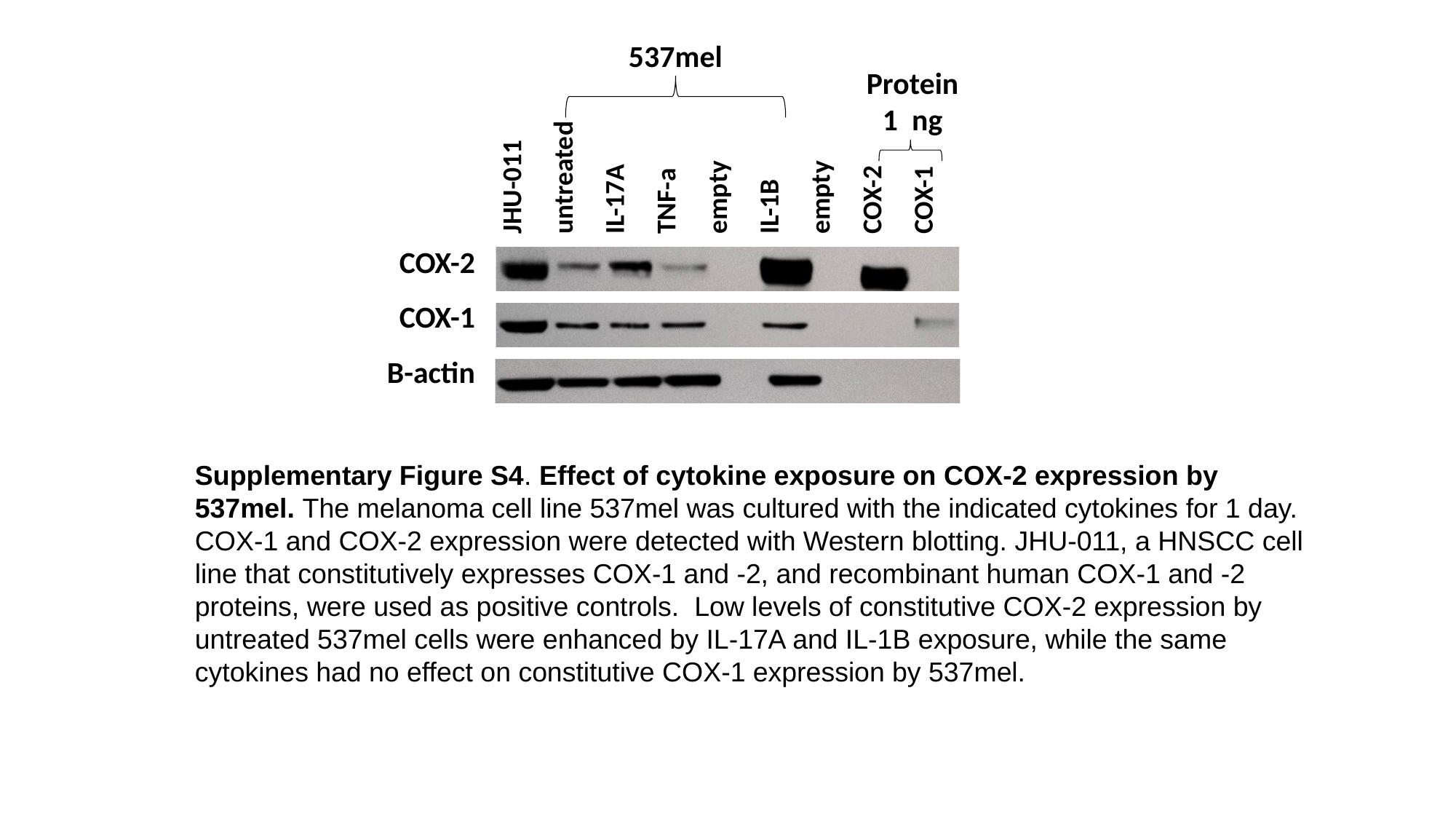

537mel
Protein
1 ng
| JHU-011 | untreated | IL-17A | TNF-a | empty | IL-1B | empty | COX-2 | COX-1 |
| --- | --- | --- | --- | --- | --- | --- | --- | --- |
| COX-2 |
| --- |
| COX-1 |
| B-actin |
Supplementary Figure S4. Effect of cytokine exposure on COX-2 expression by 537mel. The melanoma cell line 537mel was cultured with the indicated cytokines for 1 day. COX-1 and COX-2 expression were detected with Western blotting. JHU-011, a HNSCC cell line that constitutively expresses COX-1 and -2, and recombinant human COX-1 and -2 proteins, were used as positive controls. Low levels of constitutive COX-2 expression by untreated 537mel cells were enhanced by IL-17A and IL-1B exposure, while the same cytokines had no effect on constitutive COX-1 expression by 537mel.
